# Supplementary material for: EMHP: an accurate automated hole masking algorithm for single-particle cryo-EM image processing
Source: Bioinformatics. 2017 Aug 7;33(23):3824–6. doi: 10.1093/bioinformatics/btx500 (PMC5860320; doi:10.1093/bioinformatics/btx500)
Supplement: Supplementary Data [file btx500_emhp_supp_revised_sub.pdf]

Supplemental Figures and Instructions

EMHP: An accurate automated hole masking algorithm for single-particle cryo-EM image processing.

Zachary Berndsen, Charles Bowman, Haerin Jang, and Andrew B. Ward

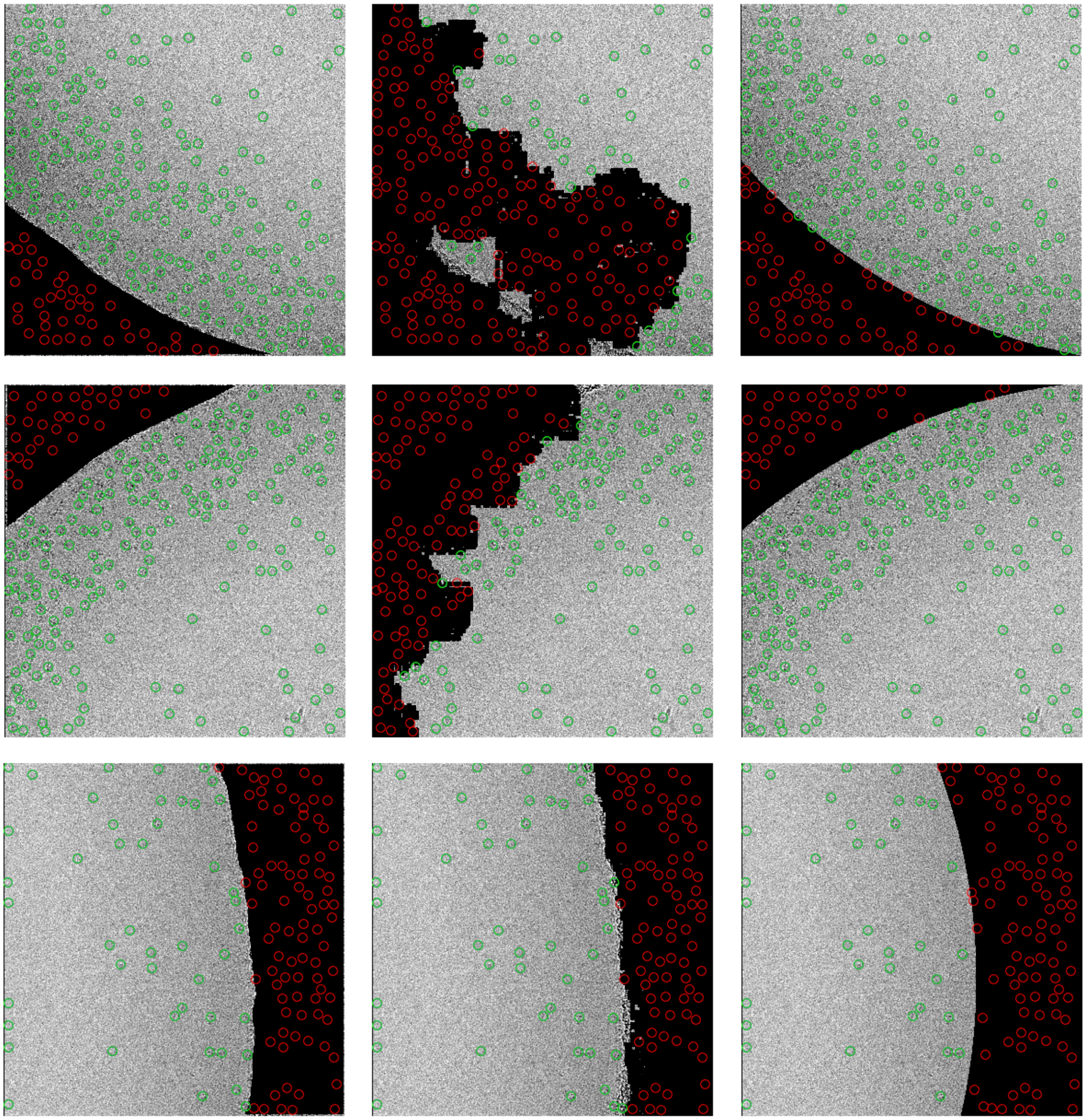

Supplemental Figure 1. Example images used for benchmarking the EMHP automasking algorithm. Each row depicts the same image filtered using: (column left) manually constructed masks used to classify particle picks, (column middle) em_hole_finder (Lander GC et al, 2009) masks, and (column right) EMHP masks. Green circles indicate picks that are included while red picks are excluded by the mask. This example does not filter edge particles. The top and center rows show over-masking by em_hole_finder, while the bottom row shows where the two algorithms perform equivalently.

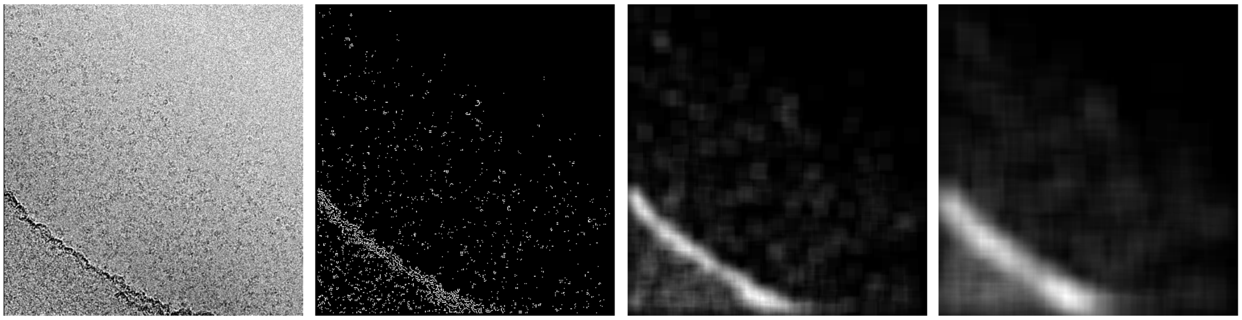

Supplemental Figure 2. Effect of changing the window size during the radial summing step. From left to right: Gaussian filter and contrast adjustments applied to input image, after applying a Sobel filter, after radial summing with window of 10 pixels, after radial summing with window of 20 pixels.

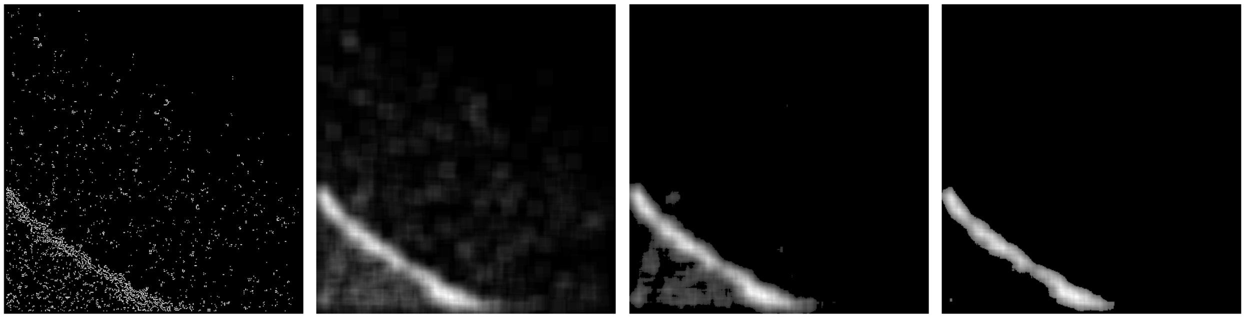

Supplemental Figure 3. Effect of changing the parameter threshold1. From left to right: Image after applying a Sobel filter, after radial summing with window of 10 pixels, after applying threshold1 of 0.2, after applying threshold1 of 0.4.

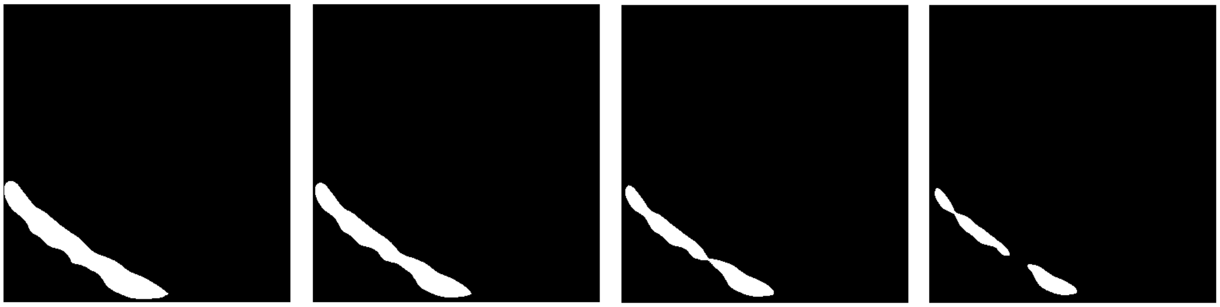

Supplemental Figure 4. Effect of changing the parameter threshold2. The input image was processed with default parameters prior the second thresholding step. From left to right: after applying threshold2 of 0.4, 0.5, 0.6, and 0.7.

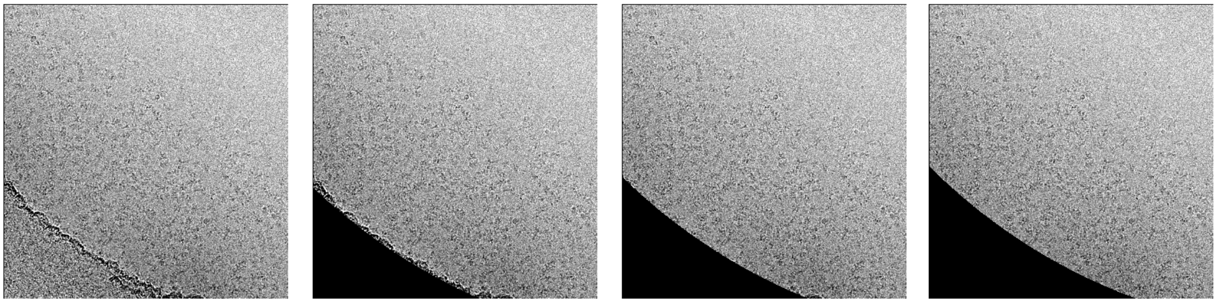

Supplemental Figure 5. Effect of mask shifting via pixel_shift parameter. From left to right: image with no mask, image with calculated mask, image with mask shift of 100px, image with mask shift of 200px, image with mask shift of 300px. Mask shifting is performed by shrinking the radius of the initially calculated circular mask, and as such results in a shift towards the center of the hole.

Download and Installation Instructions

1. License Information

EMHP is provided for your free use under the Apache License, Version 2.0. A copy of the license and more information can be found at http://www.apache.org/licenses/LICENSE-2.0

Parts of the Appion/Leginon image parsing framework have been included in this package under the Apache License, Version 2.0. More information on Appion and Leginon can be found at http://nramm.nysbc.org/software/.

2. Downloading the Software

EMHP is available at https://bitbucket.org/chazbot/emhp and can be downloaded either manually or via Git. To install the software via Git, execute the following command from within the directory you wish to install EMHP:

```
git clone https://chazbot@bitbucket.org/chazbot/emhp.git
```

After cloning the repository or downloading the files to a folder, you will need to install other python dependencies as described below before executing the programs.

Portions of the Appion/Leginon framework used for parsing .mrc images are included with this code distribution for your convenience (arraystats.py, mrc.py, resultcache.py, and weakattr.py), if you already have a working Appion/Leginon install available, you will not need the local copies and can instead depend on the libraries installed with Appion/Leginon.

3. Installing Dependencies

For EMHP's GUI functionalities to work, your version of python must have Tkinter support. Tkinter is not required for the automatic masker or pick file masker, only the assessor and manual masker.

EMHP has a few other dependencies that are not bundled that can be installed easily via python's pip functionality. Install all of them at once by executing the following command from your EMHP installation directory:

```
pip install requirements.txt
```

The dependencies are also listed here with their lowest tested versions for your convenience. Different versions may work, but these are the versions with which we locally test and deploy.

- PIL (via Pillow) tested > v3.2.0: https://python-pillow.org/
- SciPy tested > v0.17.0: https://www.scipy.org/
- NumPy tested > v1.11.0: http://www.numpy.org/
- Skimage tested > v0.12.3: http://scikit-image.org/

Usage Guide

1. File Formats

This guide assumes that you have cryo-EM images and particle pick coordinates ready to go. Images should be in .mrc format. Particle coordinates should be in one of three formats:

- Text X/Y coordinates – Text x/y coordinates are a plain text file with two columns separated by a tab character. The columns should contain the numerical x/y coordinates respectively. This is also the output format for picks in EMHP.
- Appion .pik files – These are plain text files generated by the Appion DoG Picker or Template Picker workflow. They usually contain multiple columns with extra information. EMHP can parse these files if the x/y pick coordinates are in columns two and three of the file. (which is default behavior)
- RELION .star files – These are plain text files generated by RELION's autopick function. EMHP will parse these files if the x/y pick coordinates are in the first and second column of the file. (which is default behavior) The .star file header is ignored.

Please contact us to request support for additional file types or formats.

2. Example Workflow

A typical workflow with EMHP involves the following:

- Assess images with EMHP_assess.py
- Run EMHP_auto.py on a small subset of EMHP_auto_mask.txt to optimize run parameters
- Run EMHP_auto.py on EMHP_auto_mask.txt.
- View the summary jpg images to pick out masks that are not correct.
- Add incorrect automask images to EMHP_hand_mask.txt.
- Run EMHP_manual.py on EMHP_hand_mask.txt.
- Run EMHP_picks.py on EMHP_no_mask.txt with a proper null mask.
- Copy or link images and filtered pick files to a processing directory.
- Use EMHP_keep_all.txt to feed images into single particle processing.

3. Data Setup and Considerations

When running the EMHP workflow, consistency is important. Avoid moving your images once processing has started, otherwise text files will need to be modified accordingly. Typically, we will execute the workflow in the same directory as the images themselves. Then, after processing, images and picks are moved or linked to processing directories for single particle analysis.

4. Assessing Images with EMHP_assess.py

EMHP_assess.py is a lightweight image assessor that is built using the TKinter python framework. It provides a simple interface for assessing image category, outputting a list of text files that are useful inputs for other programs and scripts.

This program requires a GUI, and should either be run locally on your processing machine, or connected to your processing nodes using window forwarding. It uses minimal resources, and as such should be safe to run on cluster head nodes or shared entry points.

The required input for the image assessor is a list of images to assess. Create this file by listing the micrographs you wish to assess into a text file.

```
ls *.mrc > images_all.txt
```

The paths to the images should either be relative to the directory from which you run the script, or absolute paths on the filesystem. Once the file list is complete, you are ready to launch! Execute the following command to start the assessor with default parameters:

```
python EMHP_assess.py --image_list=images_all.txt
```

The full listing of parameters for the script is as follows:

- Required:
  - Use **--image_list=path** to point to your list of images as described above.
- Optional:
  - Use **--scale=proportion** to adjust the size of the displayed micrograph for your screen based on a scale factor of the original image (default is .25)
  - Use the **--filter** flag to display the filtered image in the viewer by default.
  - Use **--resume** to pick up where you left off. Trying to resume a run without using this flag can cause loss of progress!
  - Use **--log_path=path** to store log files somewhere else.

After launching the script, a window will pop up showing a micrograph and assessment choices

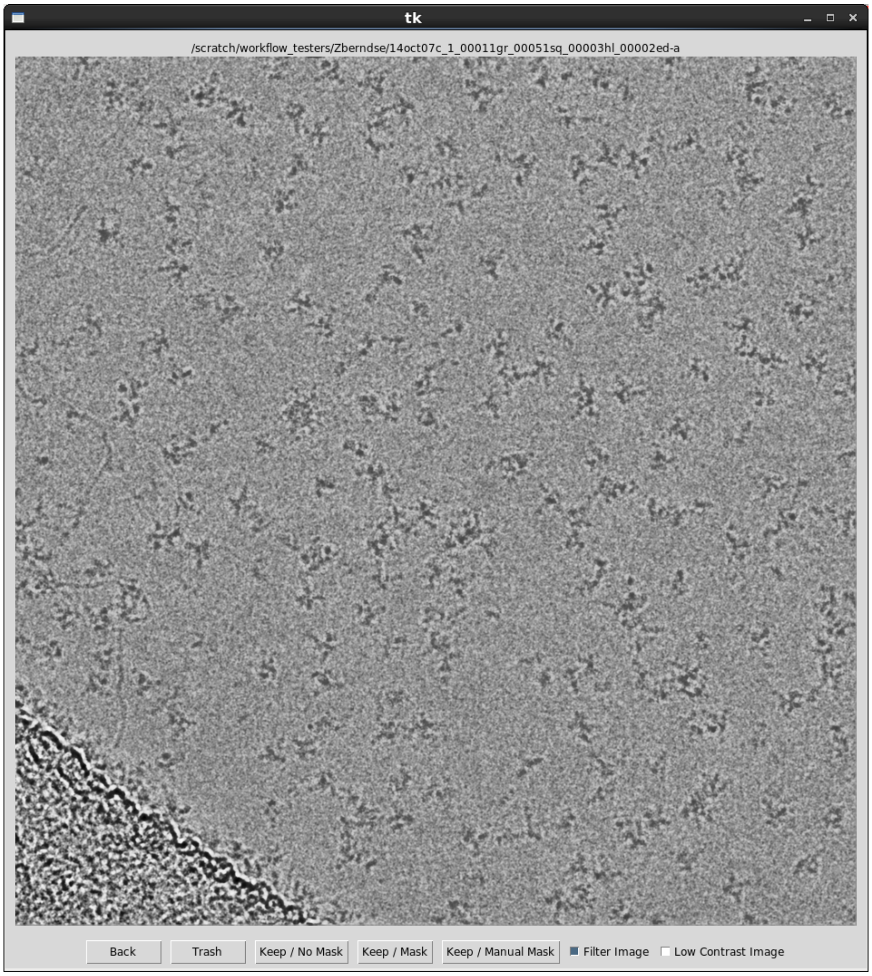

The filter option can be toggled within the GUI, or flagged as on by default. The default filter option is a simple contrast stretch. The “Low Contrast Image” checkbox in the bottom right will apply a histogram equalization to the image.

Examples of the effects of image filtering are shown below.

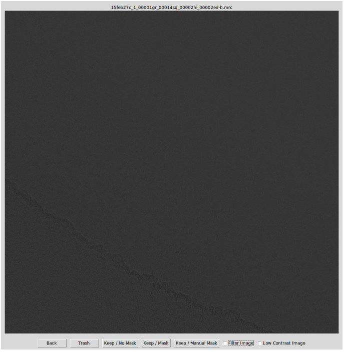

No Filter

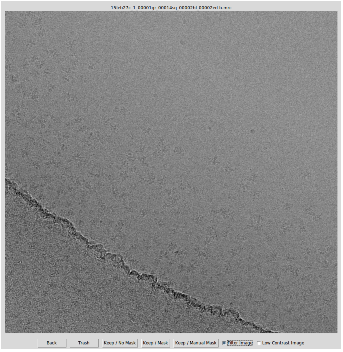

Contrast Stretching

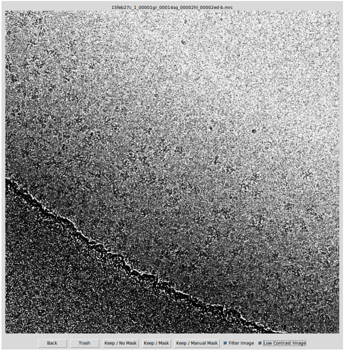

Histogram Equalization

While running the assessor, use the following controls to advance, or the buttons on the window. There is a toggle switch next to the buttons that applies a contrast filter to the images to assist in visualizing particles. For images with even lower contrast, enable the secondary filter to help see more in the images (although you might just want to trash them). Please be patient when clicking the filter button as it can take a couple seconds to re-render.

- Right Arrow - Keep image and mask
- Up Arrow - Keep image without masking
- M - Keep image and manually mask
- Down Arrow - Reject image
- Left Arrow - Go back an image

After image assessment, several text files are created based on your assessment selections. Use these in downstream processes:

- EMHP_auto_mask.txt
- EMHP_hand_mask.txt
- EMHP_no_mask.txt
- EMHP_trash.txt
- EMHP_keep_all.txt

5. **Automatic Masking using EMHP_auto.py**

EMHP_auto.py masks carbon hole edges out of electron micrographs by operating on the simple assumption that the holes we are masking are circular holes, and as such fits a circular mask over each hole based on your input parameters. This is particularly sensitive to input parameters - be sure that your hole diameter and apix are correct!

EMHP_auto.py has a built-in feature for filtering out picks based on already completed pick runs. Add the pick related flags as noted below or use the standalone pick filtering command (EMHP_picks.py) to filter picks later. The script can filter picks from many different sources. **Picks must be present in the same directory as images and have the same base name!**

Take into consideration others when running EMHP_auto.py. While it is not particularly resource-intensive, multiple masking runs at once could consume significant resources on a head node or shared system and slow it down for everyone. To take advantage of the full threading potential of the program, it is recommended to run this script either on a single compute node or your local workstation.

Execute the following command to run the automasker with default parameters:

```
python EMHP_auto.py --image_list="EMHP_auto_mask.txt" --
hole_diameter=X --apix=X.XX
```

The full listing of parameters for the script is as follows:

- **Required for Masking:**
  - Use **--image_list=file** to point to a text file containing a list of your image names, one per line.
  - Use **--hole_diameter=x** to specify grid hole size in um (typically 20000 or 12000)
  - Use **--apix=x.xx** to specify the angstrom per pixel measurement of the images you are inputting. This *must* be constant among all micrographs
- **Required for Filtering Picks:**
  - Use **--filter_picks=string** to specify the suffix of the pick files for your images to be processed. For example the filename:

    Image01.mrc

    Image01.a.pik

    You would include use everything after the basename: ".a.pik"
  - Use exactly one of the following to denote pick file format:

    --raw - Raw x/y format text pick files

    --appion_DoG - DoG picks from Appion

    --appion_template - Template picks from Appion

    --raw_relion - .star picks from RELION
  - Use **--box=x** to specify desired box size (used for edge filters)
- **Optional Arguments:**
  - Use **--write_ jpg** to write out a summary image
  - Use **--log_path=path** to point to the path to put log files
  - Use **--sigma=x (default 5)** to set the diameter of the initial Gaussian Filter
  - Use **--smoothing_radius=x (10)** to increase or decrease radius of radial sum
  - Use **--threshold1=0.0<->1.0 (.2)** to adjust first threshold cutoff
  - Use **--threshold2=0.0<->1.0 (.6)** to adjust second threshold cutoff
  - Use **--pixel_shift=x (100)** to define the compensatory shift of the hole
  - Use **--multi=X** to run multiple threads on one machine. Tested to 16
  - Use **--resume** to resume an incomplete run

This script will output a binary mask file with the suffix "_mask.png" and optionally a filtered pick file with suffix "_picks_filtered.txt" with a summary image with the suffix "_filtered.jpg" or "_masked.jpg" depending on your initial parameters.

6. **Manual Masking using EMHP_manual.py**

EMHP_manual.py operates exactly like the EMHP_auto.py with a few extra optional arguments and none of the parameters regarding the automated masking. This program requires a GUI, and should either be run locally on your processing machine, or connected to your processing nodes using window forwarding. It uses minimal resources, and as such should be safe to run on cluster head nodes or shared entry points.

Execute the following command to run manual masker with default parameters:

```
python EMHP_manual.py --image_list="EMHP_hand_mask.txt" --
hole_diameter=X --apix=X.XX
```

The full listing of parameters for the script is as follows:

- **Required for Masking:**
  - Use **--image_list=file** to point to a text file containing a list of your image names, one per line.
  - Use **--hole_diameter=x** to specify grid hole size in um (typically 20000 or 12000)
  - Use **--apix=x.xx** to specify the angstrom per pixel measurement of the images you are inputting. This *must* be constant among all micrographs
- **Required for Filtering Picks:**
  - Use **--filter_picks=string** to specify the suffix of the pick files for your images to be processed. For example the filename:

    Image01.mrc

    Image01.a.pik

    Include use everything after the basename: ".a.pik"
  - Use exactly one of the following to denote pick file format:

    --raw - Raw x/y format text pick files

    --appion_DoG - DoG picks from Appion

    --appion_template - Template picks from Appion

    --raw_relion - .star picks from RELION
  - Use **--box=x** to specify your desired box size (used for edge filters)
- **Optional Arguments:**
  - Use **--write_ jpg** to write out a summary image

- Use **--log_path=path** to point to the path to put log files
- Use **--resume** to resume an incomplete run
- Use **--low_contrast** to apply an extra filter for low contrast images
- Use **--scale=proportion** to adjust the size of the displayed micrograph for your screen based on a scale factor of the original image (default is .25)

When you run the manual masker, it will take a small amount to preprocess and load the image, and then it presents a simple GUI:

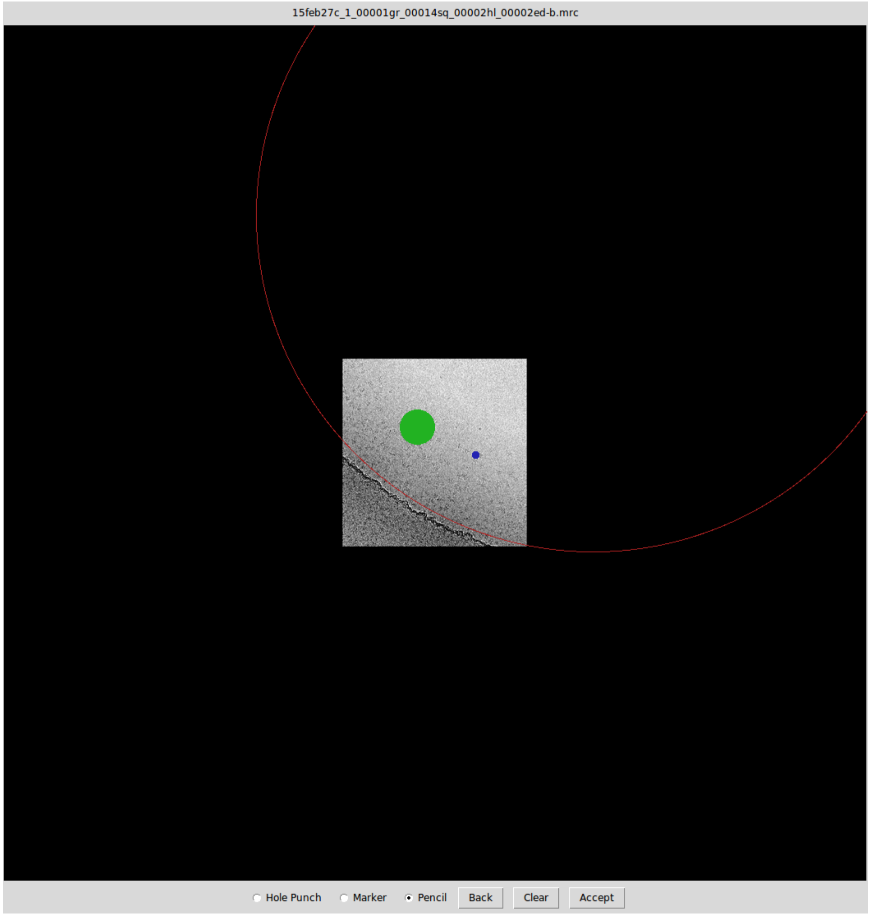

As a default, clicking anywhere on the screen will draw a red circle that represents the exact size of the holes based on your input arguments. Clicking on the radio buttons in the bottom left allow switches between the “Hole Punch”, “Marker”, and “Pencil” settings, which are the red, green, and blue circles respectively. Clicking accept after drawing any number of circles will draw them onto a mask, save the image, and filter picks. Click the other buttons to go back or clear the canvas. Multiple pencil or marker points may be present, but only one hole punch may be used. Please note that adding many small circles can slow down the mask generation significantly.

This script will output a binary mask file with the suffix "_mask.png" and optionally a filtered pick file with suffix "_picks_filtered.txt" with a summary image with the suffix "_picks_filtered.jpg".

7. **Applying Masks using EMHP_picks.py**

   EMHP_picks.py takes particle coordinate files and filters them based on binary mask images.

   Execute the following command to run manual masker with default parameters:

   ```
   python ~bowman/emhp/EMHP_picks.py --
   image_list="EMHP_no_mask.txt" --mask_suffix="_mask.png" --
   filter_picks=".a.pik" --appion_DoG --box=256
   ```

   The full listing of parameters for the script is as follows:

   - **Required Arguments:**
     - Use **--image_list=file** to point to a text file containing a list of your image names, one per line.
     - Use **--filter_picks=string** to specify the suffix of the pick files for your images to be processed. For example the filename:

       Image01.mrc

       Image01.a.pik

       Include use everything after the basename: ".a.pik"
     - Use exactly one of the following to denote pick file format:

       --raw - Raw x/y format text pick files

       --appion_DoG - DoG picks from Appion

       --appion_template - Template picks from Appion

       --raw_relion - .star picks from RELION
     - Use **--box=x** to specify your desired box size (used for edge filters)
   - **Optional Arguments:**
     - Use **--write_ jpg** to write out a summary image
     - Use **--log_path=path** to point to the path to put log files
     - Use **--resume** to resume an incomplete run

This script will output filtered pick files with suffix "_picks_filtered.txt" and optionally a summary image with the suffix "_filtered.jpg".

8. **Data Wrap-Up and Next Steps**

   After completing the EMHP workflow, you are left with an image list, masks, and filtered pick files. Move these files to any directory to continue your single particle processing.
